# Profilaxia de tromboembolismo venoso na gestação

**DOI:** 10.1590/1677-5449.006616

**Published:** 2016

**Authors:** André Luiz Malavasi Longo de Oliveira, Marcos Arêas Marques

**Affiliations:** 1 Centro de Referência da Saúde da Mulher do Estado de São Paulo, São Paulo, SP, Brasil.; 2 Universidade do Estado do Rio de Janeiro – UERJ, Hospital Universitário Pedro Ernesto – HUPE, Angiologia, Rio de Janeiro, RJ, Brasil.

**Keywords:** trombose venosa, profilaxia, gravidez

## Abstract

O tromboembolismo venoso é importante causa de morbidade e mortalidade obstétrica. Durante a gestação, o risco de sua ocorrência aumenta entre cinco e dez vezes quando comparado ao de mulheres não gestantes de mesma idade. Associado a esse fato, a gestante apresenta algumas limitações para o diagnóstico clínico (alta frequência de dor e edema nos membros inferiores), ecográfico (menor sensibilidade e especificidade no diagnóstico de trombose venosa de ilíaca com a evolução da gestação) e laboratorial (o D-dímero apresenta aumento progressivo no decorrer da gravidez). Uma estratificação criteriosa de risco de tromboembolismo venoso de cada mulher antes da gestação pode diminuir a incidência dessa doença, frequente e de difícil diagnóstico na gravidez, e suas complicações.

## INTRODUÇÃO

O parto, ao longo da história, sempre foi associado ao risco de morte. Com a evolução dos cuidados hospitalares, as intervenções médicas conseguiram reduzir as taxas de óbitos maternos e, em países que controlaram as causas clássicas de morte materna direta, como infecção puerperal, eclampsia e hemorragia, o tromboembolismo venoso (TEV) desponta como a principal delas^1^^,^ 2. Na sua forma mais letal, a embolia pulmonar (EP), o TEV apresenta uma grande barreira que dificulta o seu diagnóstico durante a gestação, causada em parte pela limitação ao uso de métodos de imagem que dependem de radiação^3^^-^ 5.

A gestante apresenta os três componentes etiopatogênicos da tríade de Virchow: a) estase, devido à compressão das veias cava e ilíaca comum esquerda pelo útero gravídico e à diminuição do tônus venoso por causa da ação miorrelaxante da progesterona; b) hipercoagulabilidade, secundária à indução da síntese hepática dos fatores VII, VIII e X de coagulação pelo estriol placentário, aumento do fibrinogênio e do inibidor do ativador do plasminogênio tipo I e II, e diminuição da síntese de proteína S; c) lesão endotelial, que ocorre na nidação, remodelação endovascular das artérias uteroespiraladas e com a dequitação^6^.

Durante a gestação, o risco de TEV aumenta de cinco a dez vezes, podendo chegar a 20 vezes no puerpério, quando comparado ao de mulheres não gestantes de mesma idade^6^^-^ 8. Após esse período, sua frequência diminui rapidamente, apesar do risco residual que persiste por até 12 semanas pós parto^9^.

A trombose venosa profunda (TVP) de membros inferiores é responsável por 75 a 80% dos episódios de TEV na gestação^6^. Aproximadamente dois terços das TVPs ocorrem no período antenatal e distribuem-se igualmente nos três trimestres. Entretanto, de 43 a 60% dos episódios de EP ocorrem nas primeiras 6 semanas do puerpério^10^^,^ 11. Nas gestantes as TVPs predominam ainda mais no membro inferior esquerdo (90% versus 55%) e no segmento íleo-femoral (72% versus 9%), quando comparadas às não gestantes. Esse fato pode ser explicado pela acentuação da compressão da veia ilíaca comum esquerda pela artéria ilíaca comum direita contra a quinta vértebra lombar, causada pelo útero gravídico^6^.

A prevalência do TEV é de 0,5 a 2,2 casos para cada 1.000 partos, dependendo da população estudada^7^^,^ 11 ^-^ 16. A incidência absoluta de TEV na gestação e puerpério foi de 107 por 100.000 mulheres-ano no Reino Unido (RU)^17^ e de 175 por 100.000 mulheres-ano na Dinamarca e no Canadá^18^^,^ 19. No Brasil, não há dados oficiais sobre a mortalidade materna por TEV^20^. A EP permanece como a principal causa de morte materna direta no RU; porém, houve uma queda significativa de mortalidade materna por EP no parto vaginal (de 1,56 por 100.000 partos em 2003-2005 para 0,70 por 100.000 partos em 2006-2008). Isso ocorreu devido à aplicação da primeira versão (2004), das diretrizes do *Royal College of Obstetricians and Gynaecologists* (RCOG) para redução do risco de TEV durante a gestação e o puerpério^21^^,^ 22. A prevenção do TEV na gestação, através de diretrizes, levando em conta os fatores de risco presentes, e a consequente instituição de profilaxia mecânica e/ou farmacológica, é a melhor estratégia para reduzir essa nefasta intercorrência^3^^-^ 5 ^,^ 23.

## METODOLOGIA

Trata-se de um estudo de revisão de literatura, considerando as publicações disponíveis na base de dados bibliográficos PUBMED no período de 2011 a 2016. A pesquisa constou das seguintes palavras-chave: *venous thrombosis, deep venous thrombosis, superficial venous thrombosis, venous thrombosis prophylaxis, treatment venous thromboembolism, disease pregnancy, pregnancy outcome, thrombophilia pregnancy* e *maternal mortality*. O critério de inclusão de artigos no estudo foi de trabalhos que contemplassem pelo menos uma das palavras-chave.

## RESULTADOS E DISCUSSÃO

### Fatores de risco

Estima-se que de 79 a 89% das gestantes que morrem por EP apresentam ao menos um fator de risco identificável^21^^-^ 24. A cesariana é um fator de risco significativo^11^^,^ 25 ^,^ 26, porém mulheres submetidas a parto vaginal estão também sob risco^21^. O TEV prévio e a presença de trombofilia previamente diagnosticada são dois fatores de risco de TEV em gestantes que podem ser identificados antes da gravidez durante a anamnese^19^^,^ 27 ^,^ 28. Estudos relatam que as trombofilias hereditárias são observadas em 20 a 50% dos casos de ocorrência de TEV na gestação^24^^,^ 29. Nas gestantes com TEV prévio, o risco de recorrência é 24,8 vezes maior^24^.

#### Obesidade

A obesidade é um importante fator de risco para TEV na gestação^11^^,^ 15 ^,^ 30 ^-^ 32 e esse risco cresce à medida que o índice de massa corporal (IMC) aumenta^33^. A obesidade (IMC > 30 kg/m^2^) é associada ao aumento de 14,9 vezes de risco de EP e TVP^32^. O sobrepeso materno (IMC entre 25 e 29,9 kg/m^2^) é um fator de risco muito comum, porém fraco, para TEV relacionado à gestação^24^. Entre as gestantes que morreram de EP no RU entre 2003 e 2008, a proporção de obesas (IMC de 30 kg/m^2^ ou mais) pode chegar a 60%^21^^,^ 22.

#### Idade

Dados extraídos de estudos do tipo caso-controle sugerem aumento de risco de duas vezes para mulheres com mais de 35 anos^14^^,^ 15 ^,^ 19. Em estudo conduzido no RU em que se utilizou uma coorte ampla de mulheres fora da gestação, aquelas com idade entre 35 e 44 anos apresentaram risco 50% maior de TEV quando comparadas com aquelas entre 25 e 34 anos. A ocorrência de TEV não aumentou com a idade no período anteparto; contudo, mulheres em fase puerperal, entre 35 e 44 anos, apresentaram risco 70% superior quando comparadas àquelas entre 25 e 34 anos (o que corresponde a aumento de risco absoluto de 1,6 por 1.000 pessoas-anos)^17^. Um estudo coreano similar observou que o aumento da faixa etária não se correlacionou a aumento do risco de TEV^25^. De modo geral, considera-se a idade de 35 anos ou mais como fator de risco antenatal e puerperal^24^.

#### Imobilidade e viagens de longa distância

Os dados relacionados à imobilidade e viagens de longa distância em gestantes são limitados, sendo necessária a extrapolação de estudos de populações não gestantes^34^^-^ 36. As diretrizes relativas a cuidados antenatais do Instituto Nacional de Excelência em Saúde e Cuidados (National Institute of Health and Care Excellence, NICE)^35^ e as recomendações do RCOG sobre viagens aéreas durante a gestação^37^ estabelecem que voos com duração superior a 4 horas aumentam o risco de TEV. Estudo caso-controle norueguês apontou aumento do risco de TEV em gestantes com IMC > 25 kg/m^2^ e imobilização anteparto (definida como restrição ao leito por tempo igual ou superior a 1 semana antes do parto ou antes do diagnóstico de TEV), mostrando efeito multiplicador sobre o risco de TEV anteparto e pós-parto (risco: 40,1 e 62,3 respectivamente)^11^.

#### Admissão hospitalar

A admissão hospitalar durante a gravidez é associada a aumento de 18 vezes no risco de TEV em comparação ao risco basal fora do hospital, e o risco permanece elevado após o parto, seis vezes maior, nos 28 dias seguintes. Na internação hospitalar, o risco é maior no terceiro trimestre de gravidez e em mulheres acima de 35 anos^38^.

#### Outros fatores de risco

Algumas comorbidades têm sido associadas ao aumento do risco de TEV durante a gestação, entre elas estão: doença intestinal inflamatória^39^, infecção do trato urinário^24^, lúpus eritematoso sistêmico, cardiopatias^19^, hipertensão arterial sistêmica induzida pela gestação ou pré-eclampsia^25^^,^ 27 e cirurgia antenatal não obstétrica^40^.

Na análise de dados de 1.475.301 altas em maternidades escocesas, Kane et al.^27^ encontraram fatores de risco associados ao TEV que incluíam três ou mais gestações anteriores, hemorragia obstétrica e pré-eclampsia.

A hiperêmese aumenta o risco de TEV pós-natal em 4,4 vezes^19^. A correta utilização dessas informações tem profundas implicações para obstetras, já que muitos eventos tromboembólicos são fatais e ocorrem no primeiro trimestre, frequentemente antes do agendamento da primeira consulta de pré-natal, quando deveria ser instituída a profilaxia antenatal^13^^,^ 21 ^,^ 24 ^,^ 41 ^,^ 42. Outros fatores de risco para o TEV e os respectivos riscos relativos estão listados na Tabela 1.

**Tabela 1:** Fatores de risco de TEV na gestação e risco relativo (RR) associado.

Fator de risco	RR
TEV prévio	24,8
Idade > 35 anos	1,3
Obesidade	2,65
IMC > 30 kg/m^2^	5,3
IMC > 25 kg/m^2^	1,8
Ganho de peso > 21 kg durante a gestação	1,6
Multiparidade	4,03
Tabagismo antenatal (10-30 cigarros/dia)	2,1
Tabagismo pós-natal (10-30 cigarros/dia)	3,4
Tabagismo na gestação	2,7
Anemia falciforme	6,7
Cardiopatia	7,1
Lúpus eritematoso sistêmico	8,7
Anemia	2,6
Veias varicosas	2,4
Imobilidade	7,7
Pré-eclampsia	3,1
Hiperêmese	4,4
Fertilização *in vivo*	4,2
Gestação gemelar	2,6
Gestação múltipla	4,2
Parto pré-maturo (< 37 semanas de gestação)	2,4
Natimorto	6,24
Hemorragia anteparto	2,3
Cesariana de emergência	2,7
Cesariana eletiva	1,3
Hemorragia pós-parto > 1 L	4,1
Hemorragia pós-parto > 1 L + cirurgia	12
Infecção pós-parto	4,1
Cesariana + infecção pós-parto	6,2
Transfusão	7,6

TEV: tromboembolismo venoso; RR: risco relativo; IMC: índice de massa corpórea.

### Profilaxia

A estratificação de risco de TEV na gestação baseia-se na avaliação de cada paciente e deve ser realizada em todas as mulheres antes da gestação e logo que engravidam, recomendando-se repeti-la ao longo do pré-natal, diante de eventual surgimento de novos fatores de riscos. As preferências e as considerações das gestantes devem ser levadas em conta no momento da escolha da tromboprofilaxia^43^.

Abaixo, foram sintetizadas as diretrizes das entidades mais relevantes na área de diagnóstico, profilaxia e tratamento do TEV na gestação: *American College of Obstetricians and Gynaecologists* (ACOG)^44^, *Society of Obstetricians and Gynaecologists of Canada* (SOGC)^43^, RCOG^45^ e Ame*rican College of Chest Physicians* (ACCP)^46^. A Tabela 2 apresenta a dosagem das heparinas sugerida para profilaxia de TEV em gestantes de acordo com SOCG^43^.

**Tabela 2:** Doses sugeridas de HBPM e HNF na profilaxia do TEV relacionados à gestação pela SOCG^43^.

**Dose profilática de HNF**
5.000 UI SC duas vezes ao dia
**Dose intermediária de HNF**
10.000 UI SC duas vezes ao dia
**Dose profilática de HBPM**
Dalteparina 5.000 UI uma vez ao dia
Enoxaparina 40 mg uma vez ao dia
**Dose intermediária de HBPM**
Dalteparina 5.000 UI duas vezes ao dia ou 10.000 UI uma vez ao dia
Enoxaparina 80 mg uma vez ao dia ou 40 mg duas vezes ao dia

HBPM: heparina de baixo peso molecular; HNF: heparina não fracionada; SGC: Sociedade de Ginecologia e Obstetrícia do Canadá; SC: subcutâneo.

#### Prevenção de recorrência de TEV

##### Episódio único de TEV sem uso de anticoagulação de longa duração e com trombofilia conhecida

###### Heterozigose do fator V de Leiden ou mutação do gene 20210 da protrombina

Anteparto

ACOG: dose profilática ou intermediária de heparina de baixo peso molecular (HBPM), dose profilática de heparina não fracionada (HNF), ou observação clínica^44^.

SOGC: dose profilática de HNF ou HBPM (preferencialmente)^43^.

RCOG: dose profilática de HBPM por toda a gestação^45^.

ACCP: baixo risco de recorrência (episódio único associado a risco transitório não relacionado à gestação ou a estrógeno): observação clínica;

Risco moderado a alto (episódio único de TEV não provocado, TEV relacionado à gestação ou ao uso de estrógeno ou múltiplos TEVs não provocados) sem anticoagulação de longa duração: dose profilática ou intermediária de HBPM^46^.

Pós-parto

ACOG: dose intermediária de HBPM ou HNF ou anticoagulação com antagonistas da vitamina K (AVK) por 4 a 6 semanas^44^.

SOGC: dose profilática de HNF ou HBPM (preferencialmente) por 6 semanas^43^.

RCOG: dose profilática de HBPM ou anticoagulação com AVK^45^.

ACCP: dose profilática ou intermediária de HBPM ou anticoagulação com AVK por 6 semanas^46^.

###### Deficiência de proteína C ou S

Anteparto

ACOG: dose profilática ou intermediária de HBPM, HNF ou observação clínica^44^.

SOGC: dose profilática de HNF ou HBPM (preferencialmente)^43^.

RCOG: dose profilática de HBPM por toda a gestação^45^.

ACCP: baixo risco de recorrência: observação clínica;

Risco moderado a alto sem anticoagulação de longa duração: dose profilática ou intermediária de HBPM^46^.

Pós-parto

ACOG: anticoagulação com AVK ou dose intermediária de HBPM ou HNF por 4 a 6 semanas^44^.

SOGC: dose profilática de HNF ou HBPM (preferencialmente) por 6 semanas^43^.

RCOG: dose profilática de HBPM ou anticoagulação com AVK por 6 semanas^45^.

ACCP: dose profilática ou intermediária de HBPM por 6 semans^46^.

###### Heterozigose composta

Anteparto

ACOG: dose profilática, intermediária ou ajustada de HBPM ou HNF^44^.

SOGC: dose intermediária ou terapêutica de HNF ou HBPM (preferencialmente)^43^.

RCOG: dose profilática de HBPM^45^.

ACCP: baixo risco de recorrência de TEV: observação clínica^46^.

Pós-parto

ACOG: dose intermediária ou ajustada de HBPM, HNF ou anticoagulação com AVK por 4 a 6 semanas^44^.

SOGC: dose profilática de HNF ou HBPM (preferencialmente) por 6 semanas^43^.

RCOG: dose profilática de HBPM ou anticoagulação com AVK por pelo menos 6 semanas^45^.

ACCP: dose profilática ou intermediária de HBPM ou anticoagulação com AVK por 6 semanas^46^.

###### Deficiência de antitrombina

Anteparto

ACOG: dose profilática, intermediária ou ajustada de HBPM ou de HNF^44^.

SOGC: dose intermediária ou terapêutica de HNF ou HBPM (preferencialmente)^43^.

RCOG: manejo em conjunto com médico especialista em anticoagulação ou trombose na gestação; considerar a dosagem sérica antenatal do fator anti-Xa e a possibilidade de reposição de antitrombina no início do trabalho de parto ou antes da cesariana; se os níveis de anti-Xa forem dosados, deve-se realizar teste que não use antitrombina exógena com alvo no pico de 4 horas após a administração de 0,5 a 1,0 UI/mL: dose alta de HBPM (50, 75 ou 100% da dose plena ajustada por peso)^45^.

ACCP: baixo risco de recorrência: observação clínica;

Risco de moderado a alto sem anticoagulação de longa duração: dose profilática ou intermediária de HBPM^46^.

Pós-parto

ACOG: dose profilática ou intermediária de HBPM, HNF ou anticoagulação com AVK por 4 a 6 semanas^44^.

SOGC: dose profilática de HNF ou HBPM (preferencialmente) por 6 semanas^43^.

RCOG: HBPM, 50, 75 ou 100% da dose plena ajustada por peso por 6 semanas ou até o retorno da anticoagulação oral^45^.

ACCP: dose profilática ou intermediária de HBPM ou anticoagulação com AVK^46^.

###### Síndrome do anticorpo antifosfolipídio (SAF)

Anteparto

ACOG: anticoagulação com heparina por toda a gestação^44^.

SOGC: dose intermediária ou terapêutica de HNF ou HBPM (preferencialmente)^43^.

RCOG: manejo em conjunto com médico especialista em anticoagulação ou trombose na gestação: profilaxia com dose alta de HBPM (50, 75 ou 100% da dose plena ajustada por peso)^45^.

ACCP: baixo risco de recorrência: observação clínica;

Risco de moderado a alto de recorrência sem anticoagulação de longa duração: dose profilática ou intermediária de HBPM^46^.

Pós-parto

ACOG: 6 semanas de anticoagulação com heparina^44^.

SOGC: dose profilática de HNF ou HBPM (preferencialmente) por 6 semanas^43^.

RCOG: dose alta de HBPM (50%, 75% ou 100% da dose plena ajustada por peso) ou até o retorno da anticoagulação oral^45^.

ACCP: dose profilática ou intermediária de HBPM ou anticoagulação com AVK^46^.

##### TEV prévio associado a fator de risco transitório não relacionado a estrógeno, sem trombofilia conhecida

Anteparto

ACOG: observação clínica^44^.

SOGC: dose profilática de HNF ou HBPM (preferencialmente)^43^.

RCOG: se o TEV foi provocado por cirurgia de grande porte, sem outros fatores de risco, a tromboprofilaxia com HBPM pode ser iniciada a partir de 28 semanas, desde que não haja outros fatores de risco; se o TEV original tiver relação com fatores de risco transitórios, exceto cirurgia de grande porte, a HBPM deve ser administrada por toda a gestação^45^.

ACCP: baixo risco de recorrência: observação clínica^46^.

Pós-parto

ACOG: terapia anticoagulante pós-parto^44^.

SOGC: dose profilática de HNF ou HBPM (preferencialmente) por 6 semanas^43^.

RCOG: dose profilática de HBPM ou anticoagulação com AVK por pelo menos 6 semanas^45^.

ACCP: dose profilática ou intermediária de HBPM ou anticoagulação com AVK por 6 semanas, se não houver deficiência de proteína C ou S^46^.

##### TEV prévio associado à gestação ou ao uso de estrógeno

Anteparto

ACOG: dose profilática de HBPM ou HNF^44^.

SOGC: dose profilática de HNF ou HBPM (preferencialmente)^43^.

RCOG: tromboprofilaxia com HBPM^45^.

ACCP: risco moderado a alto de recorrência sem anticoagulação de longa duração: dose profilática ou intermediária de HBPM^46^.

Pós-parto

ACOG: terapia anticoagulante pós-parto^44^.

SOGC: dose profilática de HNF ou HBPM (preferencialmente) por 6 semanas^43^.

RCOG: profilaxia com HBPM ou anticoagulação com AVK por pelo menos 6 semanas^45^.

ACCP: dose profilática ou intermediária de HBPM ou anticoagulação com AVK, por 6 semanas, se não houver deficiência de proteína C ou S^46^.

##### TEV prévio não provocado

Anteparto

ACOG: dose profilática de HBPM ou HNF^44^.

SOGC: dose profilática de HNF ou HBPM (preferencialmente)^43^.

RCOG: dose profilática de HBPM^45^.

ACCP: risco de moderado a alto de recorrência de TEV sem anticoagulação de longa duração: dose profilática ou intermediária de HBPM^46^.

Pós-parto

ACOG: terapia anticoagulante pós-parto^44^.

SOGC: dose profilática de HNF ou HBPM (preferencialmente) por 6 semanas^43^.

RCOG: dose profilática de HBPM ou AVK por pelo menos seis semanas^45^.

ACCP: dose profilática ou intermediária de HBPM ou anticoagulação com AVK por seis semanas, se não houver deficiência de proteína C ou S^46^.

##### Dois ou mais episódios de TEV sem uso de anticoagulação de longa duração

Anteparto

ACOG: dose profilática ou terapêutica de HBPM ou HNF^44^.

SOGC: dose profilática de HNF ou HBPM (preferencialmente)^43^.

RCOG: acompanhamento com especialista em trombose na gravidez: dose alta de HBPM (50%, 75% ou 100% da dose ajustada por peso)^45^.

ACCP: risco moderado a alto de recorrência sem anticoagulação de longa duração: dose profilática ou intermediária de HBPM^46^.

Pós-parto

ACOG: anticoagulação pós-parto por 4 a 6 semanas^44^.

SOGC: dose profilática de HNF ou HBPM (preferencialmente) por 6 semanas^43^.

RCOG: dose alta de HBPM (50%, 75% ou 100% da dose plena ajustada por peso) por 6 semanas^45^.

ACCP: dose profilática ou intermediária de HBPM ou anticoagulação com AVK por 6 semanas, se não houver deficiência de proteína C ou S^46^.

##### Dois ou mais episódios de TEV com uso de anticoagulação de longa duração

Anteparto

ACOG: dose terapêutica de HBPM ou HNF^44^.

SOGC: dose profilática de HNF ou HBPM (preferencialmente)^43^.

RCOG: as mulheres devem ser alertadas sobre os riscos do uso de AVK para o feto e aconselhadas a interromper tais medicações e a mudar para HBPM assim que a gestação se confirmar (o ideal seria com 2 semanas de atraso menstrual e antes de 6 semanas de gravidez): dose alta de HBPM (50%, 75% ou 100% da dose plena ajustada por peso)^44^.

ACCP: em caso de uso de longa duração de AVK e se a paciente for candidata à substituição por HBPM, sugere-se a realização frequente de testes de gravidez e a substituição de AVK por HBPM somente quando se confirmar a gravidez. Recomenda-se dose ajustada ou 75% da dose terapêutica de HBPM^46^.

Pós-parto

ACOG: retomar a anticoagulação de longa duração^44^.

SOGC: retomar a anticoagulação de longa duração^43^.

RCOG: dose alta de HBPM (50%, 75% ou 100% da dose plena ajustada por peso) por 6 semanas ou até o retorno da anticoagulação oral. Pode-se reiniciar o uso de AVK no caso de mulheres que recebem anticoagulação de longa duração com esse agente quando o risco de sangramento se reduzir, usualmente de 5 a 7 dias pós-parto^45^.

ACCP: sugere-se a retomada da anticoagulação de longa duração em vez da administração de dose profilática de HBPM^46^.

#### Prevenção de TEV associado a cesariana

Revisão sistemática da Cochrane concluiu que não há evidências suficientes da tromboprofilaxia pós-cesariana devido ao pequeno número de estudos e a diferentes critérios de comparação^47^. Embora o risco de TEV associado à cesariana seja baixo^15^^,^ 48 ^-^ 50, quando há relação com outros fatores de risco a ocorrência de TEV passa a ser significativa e deve-se indicar a instituição de tromboprofilaxia^43^^-^ 46.

ACOG: compressão pneumática intermitente (CPI) antes da cesariana se a paciente não fizer uso de tromboprofilaxia^44^.

SOGC: recomenda-se profilaxia farmacológica no pós-parto diante das seguintes situações: TEV prévio; trombofilia de alto risco (SAF, deficiência de antitrombina, homozigose do fator V de Leiden ou mutação do gene G20210A da protrombina ou trombofilias combinadas), restrição ao leito antes do parto por 7 ou mais dias, sangramento maior que 1 L no periparto ou no pós-parto, transfusão de hemoderivados, cirurgia pós-parto e infecção no periparto ou no pós-parto^43^. Deve-se considerar o uso de profilaxia farmacológica na ocorrências de duas ou mais das seguintes situações: IMC ≥ 30 kg/m^2^ na primeira consulta pré-natal, tabagismo > 10 cigarros/dia, pré-eclampsia, restrição de crescimento fetal, placenta prévia, cesariana de emergência, sangramento maior que 1 L no periparto ou no pós-parto ou transfusão de hemoderivados, trombofilia de baixo risco (deficiência de proteínas C ou S, heterozigose do fator V de Leiden ou mutação 20210A do gene da protrombina), doença cardíaca materna, lúpus eritematoso sistêmico, anemia falciforme, doença inflamatória intestinal, varizes de membros inferiores, diabetes gestacional, parto prematuro, parto de natimorto, ou três ou mais dos seguintes fatores de risco: idade > 35 anos, paridade ≥ 2, qualquer técnica de reprodução assistida, gestação múltipla, descolamento prematuro de placenta, ruptura prematura de membranas, cesariana eletiva ou câncer materno. As mulheres com fatores de risco persistentes devem receber tromboprofilaxia no mínimo por 6 semanas pós-parto; as mulheres com fatores de risco transitórios no anteparto e intraparto devem receber tromboprofilaxia até a alta hospitalar ou até 2 semanas após o parto^43^.

RCOG: cesariana de emergência, 10 dias após o parto com dose profilática de HBPM; para todas as outras pacientes submetidas a cesariana, considerar 10 dias de HBPM em dose profilática se houver outros fatores de risco^45^.

ACCP: na ausência de fatores de risco adicionais, não utilizar profilaxia além de deambulação precoce; no caso de um fator de risco maior ou de ≥ dois fatores de risco menores (um menor se houver cesariana de emergência), sugere-se profilaxia com HBPM após o parto enquanto a paciente permanecer no hospital (se houver contraindicação de anticoagulação, usar profilaxia mecânica com meias elásticas ou CPI); no caso de risco altíssimo com fatores de risco adicionais que persistem no puerpério, combinar HBPM com meias elásticas e/ou CPI; as pacientes selecionadas de alto risco com fatores de risco adicionais que persistem no puerpério devem receber até 6 semanas de extensão de profilaxia após a alta hospitalar^46^.

## CONCLUSÃO

As recomendações sugeridas aqui dependem de variações individuais entre os pacientes e têm o intuito de informar, e não de substituir, o julgamento clínico do médico, que em última análise deve determinar o tratamento apropriado para cada indivíduo. Porém, com uma abordagem profilática apropriada, a incidência de TEV na gestante pode diminuir, evitando assim suas complicações agudas e crônicas.
