# Supplementary material for: Profilaxia de tromboembolismo venoso na gestação
Source: J Vasc Bras. 2016 Oct-Dec;15(4):293–301. [Article in Portuguese] doi: 10.1590/1677-5449.006616 (PMC5829728; doi:10.1590/1677-5449.006616)
Supplement: Translation

# Translation: Venous thromboembolism prophylaxis in pregnancy

**Authors:** André Luiz Malavasi Longo de Oliveira, Marcos Arêas Marques

**Affiliations:** 1 Centro de Referência da Saúde da Mulher do Estado de São Paulo, São Paulo, SP, Brazil.; 2 Universidade do Estado do Rio de Janeiro – UERJ, Hospital Universitário Pedro Ernesto – HUPE, Angiologia, Rio de Janeiro, RJ, Brazil.

**Keywords:** venous thrombosis, prophylaxis, pregnancy

## Abstract

Venous thromboembolism is a major cause of obstetric morbidity and mortality. During pregnancy, the risk of occurrence increases between five and ten times when compared to women of the same age who are not pregnant. Compounding this is the fact that pregnant women present certain characteristics that make diagnosis more difficult using clinical signs (high frequency of pain and swelling in the lower limbs), echographic examination (lower sensitivity and specificity for diagnosis of iliac vein thrombosis as pregnancy progresses), and laboratory findings (D-dimer levels progressively increase throughout pregnancy). Conducting careful stratification of women’s venous thromboembolism risk before pregnancy could reduce the incidence of this disease, which is frequent and difficult to diagnose during pregnancy, and of its complications.

## INTRODUCTION

Throughout history, giving birth has always been associated with a risk of death. As hospital care has improved, medical interventions have reduced maternal death rates and, in countries that control the classic direct causes of maternal death, such as puerperal infection, eclampsia, and hemorrhage, venous thromboembolism (VTE) figures as the number one cause.^1^^,^ 2 In its most lethal form, VTE, pulmonary embolism (PE) is subject to serious barriers that make diagnosis during pregnancy much less likely, which is partly the result of restrictions to the use of imaging methods that are dependent on radiation.^3^^-^ 5

Pregnant women have three of the etiopathogenic components of Virchow’s triad: a) stasis, caused by compression of the vena cava and left common iliac by the pregnant uterus and by reduced venous tone resulting from the myorelaxant action of progesterone; b) hypercoagulability, secondary to induction of hepatic synthesis of coagulation factors VII, VIII, and X by placental estriol, increased levels of fibrinogen and plasminogen activator inhibitor types I and II, and reduced synthesis of protein S; c) endothelial injury, which occurs in implantation, endovascular remodeling of the uterine spiral arteries, and placental delivery.^6^

The risk of VTE is five to 10 times greater during pregnancy and can be as much as 20 times greater during the puerperium, when compared with women of the same age who are not pregnant.^6^^-^ 8 After this period, the frequency reduces rapidly, although there is a residual risk that lasts for up to 12 weeks after delivery.^9^

Deep venous thrombosis (DVT) in lower limbs is responsible for 75 to 80% of VTE episodes during pregnancy.^6^ Approximately two thirds of DVTs occur during the prenatal period and are equally distributed across the three trimesters. However, 43 to 60% of PE events occur during the first 6 weeks of the postpartum period.^10^^,^ 11 Among pregnant women, DVTs are predominately seen in the left lower limb (90% vs. 55%) and the iliofemoral segment (72% vs. 9%), when compared with women who are not pregnant. This phenomenon can be attributed to the increased compression of the left common iliac vein by the right common iliac artery against the fifth lumbar vertebra, caused by the pregnant uterus.^6^

The prevalence of VTE is 0.5 to 2.2 cases in every 1,000 births, depending on the population studied.^7^^,^ 11 ^-^ 16 The absolute incidence of VTE during pregnancy and the puerperium was 107 per 100,000 woman-years in the United Kingdom (UK)^17^ and 175 per 100,000 woman-years in Denmark and Canada.^18^^,^ 19 In Brazil there are no official data on maternal mortality due to VTE.^20^ In the UK, PE is still the number one direct cause of maternal deaths; but there was a significant reduction in maternal mortality from PE in vaginal deliveries (from 1.56 per 100,000 births in 2003-2005 to 0.70 per 100,000 births in 2006-2008). This was the result of adoption of the first version (2004) of the Royal College of Obstetricians and Gynaecologists’(RCOG) guidelines for reduction of VTE risk during pregnancy and the puerperium.^21^^,^ 22 Prevention of VTE during pregnancy, by following guidelines that consider risk factors and indicate institution of mechanical and/or pharmacological prophylaxis is the best strategy for reducing the rate of this highly dangerous complication.^3^^-^ 5 ^,^ 23

## METHODOLOGY

This article describes a review of literature indexed on the PUBMED bibliographic database, with publication dates from 2011 to 2016. The search strategy employed the following keywords: venous thrombosis, deep venous thrombosis, superficial venous thrombosis, venous thrombosis prophylaxis, treatment venous thromboembolism, disease pregnancy, pregnancy outcome, thrombophilia pregnancy, and maternal mortality. The criterion for selecting articles for the study was those with at least one of these keywords.

## RESULTS AND DISCUSSION

### Risk factors

It is estimated that 79 to 89% of pregnant women who die because of PE exhibit at least one identifiable risk factor.^21^^-^ 24 Caesarean delivery is one significant risk factor,^11^^,^ 25 ^,^ 26 but women who deliver vaginally are also at risk.^21^ A previous VTE and a prior diagnosis of thrombophilia are two risk factors for VTE in pregnant women that can be identified from a patient history before pregnancy.^19^^,^ 27 ^,^ 28 Studies report that hereditary thrombophilias are observed in 20 to 50% of cases of VTE events in pregnancy.^24^^,^ 29 Pregnant women who have had a prior VTE are at a 24.8 times greater risk of recurrence.^24^

#### Obesity

Obesity is another important risk factor for VTE during pregnancy^11^^,^ 15 ^,^ 30 ^-^ 32 and the level of risk rises as body mass index (BMI) increases.^33^ Obesity (BMI > 30 kg/m^2^) is associated with a 14.9 times increase in the risk of PE and DVT.^32^ Maternal overweight (BMI from 25 to 29.9 kg/m^2^) is a very common, although weak, risk factor for pregnancy-related VTE.^24^ The proportion of pregnant women who died from PE in the UK between 2003 and 2008 who were obese (BMI of 30 kg/m^2^ or more) was as high as 60%.^21^^,^ 22

#### Age

Data extracted from case-control studies suggest that the risk is double for women over the age of 35.^14^^,^ 15 ^,^ 19 A study conducted in the UK with a large cohort of women who were not pregnant found that participants aged from 35 to 44 had a 50% higher risk of VTE when compared with those aged 25 to 34. Rates of prenatal VTE did not increase with age, but recently-delivered women aged 35 to 44 exhibited a 70% higher risk when compared to those aged 25 to 34 (equivalent to an increase in absolute risk of 1.6 per 1,000 person-years).^17^ A similar study conducted in Korea observed that increases in age were not correlated with increased risk of VTE.^25^ In general, it is considered that age greater than 35 years is both a prenatal and postnatal risk factor.^24^

#### Immobility and long-distance travel

There is limited data on immobility and long-distance travel in pregnant women and it is necessary to extrapolate evidence from studies of populations of women who are not pregnant.^34^^-^ 36 Guidelines published by the UK National Institute of Health and Care Excellence (NICE)^35^ and the RCOG’s recommendations on air travel during pregnancy^37^ state that flights that last longer than 4 hours increase the risk of VTE. A Norwegian case-control study indicated that there is an increased risk of VTE among pregnant women with BMI > 25 kg/m^2^ and prenatal immobilization (defined as strict bed rest for a period of 1 week or more before delivery or before diagnosis of VTE), showing the multiplication effects on risk of prenatal and postnatal VTE (risk: 40.1 and 62.3 respectively).^11^

#### Hospital admission

Hospital admission during pregnancy is associated with an 18 times greater risk of VTE compared with baseline risk for those not in hospital, and the risk remains high after delivery; six times higher up to 28 days after birth. Hospital admission risk is greatest during the third trimester of pregnancy and among women over the age of 35.^38^

#### Other risk factors

Certain comorbidities have been associated with increased risk of VTE during pregnancy, including inflammatory intestinal disease,^39^ urinary tract infection,^24^ systemic lupus erythematosus, heart diseases,^19^ systemic arterial hypertension induced by pregnancy or pre-eclampsia,^25^^,^ 27 and non- obstetric prenatal surgery.^40^

An analysis of data from 1,475,301 discharges from Scottish maternity units conducted by Kane et al.^27^ identified risk factors associated with VTE that included three or more previous pregnancies, obstetric hemorrhage, and pre-eclampsia.

Hyperemesis increases the risk of postnatal VTE by a factor of 4.4.^19^ Correct use of this information has profound implications for obstetricians, since many thromboembolic events are fatal and occur during the first trimester, often before the first prenatal consultation has been scheduled, which is when prenatal prophylaxis will be initiated.^13^^,^ 21 ^,^ 24 ^,^ 41 ^,^ 42 Other risk factors for VTE and their respective relative risks are listed in Table 1.

**Table 1:** Risk factors for VTE during pregnancy and their associated relative risks (RR).

Risk factor	RR
Previous VTE	24.8
Age > 35 years	1.3
Obesity	2.65
BMI > 30 kg/m^2^	5.3
BMI > 25 kg/m^2^	1.8
Weight gain > 21 kg during pregnancy	1.6
Multiparity	4.03
Prenatal smoking (10-30 cigarettes/day)	2.1
Postnatal smoking (10-30 cigarettes/day)	3.4
Smoking during pregnancy	2.7
Sickle-cell anemia	6.7
Heart disease	7.1
Systemic lupus erythematosus	8.7
Anemia	2.6
Varicose veins	2.4
Immobility	7.7
Pre-eclampsia	3.1
Hyperemesis	4.4
In vivo fertilization	4.2
Twin pregnancy	2.6
Multiple pregnancy	4.2
Premature delivery (< 37 weeks gestation)	2.4
Still birth	6.24
Prepartum hemorrhage	2.3
Emergency	2.7
Elective caesarean	1.3
Postpartum hemorrhage > 1 L	4.1
Postpartum hemorrhage > 1 L + surgery	12
Postpartum infection	4.1
Caesarean + postpartum infection	6.2
Transfusion	7.6

VTE: venous thromboembolism; RR: relative risk; BMI: body mass index.

### Prophylaxis

Stratification of risk of VTE during pregnancy is based on case-by-case assessment of every patient and should be conducted for all women before pregnancy and as soon as they become pregnant, and it is recommended that assessments should be repeated throughout prenatal care, as new risk factors emerge. The pregnant woman’s preferences and values should be taken into account when thromboprophylaxis is being chosen.^43^

A summary is given below of the guidelines for diagnosis, prophylaxis, and treatment of VTE during pregnancy published by the relevant medical associations: the American College of Obstetricians and Gynaecologists (ACOG),^44^ the Society of Obstetricians and Gynaecologists of Canada (SOGC),^43^ the UK’s RCOG,^45^ and the American College of Chest Physicians (ACCP).^46^ Table 2 lists the heparin dosages suggested by the SOGC for prophylaxis against VTE in pregnant women.^43^

**Table 2:** Dosages of LMWH and UFH for prophylaxis of VTE related to pregnancy suggested b y the SOCG^43^.

**Prophylactic dose of UFH**
5,000 UI SC twice a day
**Intermediate dose of UFH**
10,000 UI SC twice a day
**Prophylactic dose of LMWH**
Dalteparin: 5,000 UI once a day
Enoxaparin: 40 mg once a day
**Intermediate dose of LMWH**
Dalteparin: 5,000 UI twice a day or 10,000 UI once a day
Enoxaparin: 80 mg once a day or 40 mg twice a day

LMWH: low-molecular-weight heparin; UFH: unfractionated heparin; VTE: venous thromboembolism; SOCG: The Society of Obstetricians and Gynaecologists of Canada; SC: subcutaneous.

#### Prevention of VTE recurrence

##### Single VTE without use of long-term anticoagulation and with known thrombophilia

###### Heterozygosity for factor V Leiden or mutation of the 20210 prothrombin gene

Antepartum

ACOG: prophylactic or intermediate doses of low molecular weight heparin (LMWH), prophylactic doses of unfractionated heparin (UFH), or clinical observation.^44^

SOGC: prophylactic doses of UFH or LMWH (preferable).^43^

RCOG: prophylactic doses of LMWH for entire pregnancy.^45^

ACCP: low risk of recurrence (single episode associated with transitory risk unrelated to pregnancy or estrogen): clinical observation;

Moderate to high risk (single unprovoked VTE episode, VTE related to pregnancy or to use of estrogen, or multiple unprovoked VTEs) without use of long-term anticoagulation: prophylactic or intermediate doses of LMWH.^46^

Postpartum

ACOG: intermediate doses of LMWH or UFH or anticoagulation with vitamin K antagonists (VKA) for 4 to 6 weeks.^44^

SOGC: prophylactic doses of UFH or LMWH (preferable) for 6 weeks.^43^

RCOG: prophylactic doses of LMWH or anticoagulation with VKA.^45^

ACCP: prophylactic or intermediate doses of LMWH or anticoagulation with VKA for 6 weeks.^46^

###### Protein C or S deficiency

Antepartum

ACOG: prophylactic or intermediate doses of LMWH, UFH or clinical observation.^44^

SOGC: prophylactic doses of UFH or LMWH (preferable).^43^

RCOG: prophylactic doses of LMWH for entire pregnancy.^45^

ACCP: low risk of recurrence: clinical observation;

Moderate to high risk without use of long-term anticoagulation: prophylactic or intermediate doses of LMWH.^46^

Postpartum

ACOG: anticoagulation with VKA or intermediate doses of LMWH or UFH for 4 to 6 weeks.^44^

SOGC: prophylactic doses of UFH or LMWH (preferable) for 6 weeks.^43^

RCOG: prophylactic doses of LMWH or anticoagulation with VKA for 6 weeks.^45^

ACCP: prophylactic or intermediate doses of LMWH for 6 weeks.^46^

###### Combined heterozygosity

Antepartum

ACOG: prophylactic, intermediate, or adjusted doses of LMWH or UFH.^44^

SOGC: intermediate or therapeutic doses of UFH or LMWH (preferable).^43^

RCOG: prophylactic doses of LMWH.^45^

ACCP: low risk of recurrence of VTE: clinical observation.^46^

Postpartum

ACOG: intermediate or adjusted doses of LMWH, UFH or anticoagulation with VKA for 4 to 6 weeks.^44^

SOGC: prophylactic doses of UFH or LMWH (preferable) for 6 weeks.^43^

RCOG: prophylactic doses of LMWH or anticoagulation with VKA for at least 6 weeks.^45^

ACCP: prophylactic or intermediate doses of LMWH or anticoagulation with VKA for 6 weeks.^46^

###### Antithrombin deficiency

Antepartum

ACOG: prophylactic, intermediate, or adjusted doses of LMWH or UFH.^44^

SOGC: intermediate or therapeutic doses of UFH or LMWH (preferable).^43^

RCOG: management should involve a physician who is a specialist in anticoagulation or thrombosis during pregnancy; consider serial prenatal assays of anti-Xa factor and evaluate the possibility of antithrombin replacement at the start of labor or before a caesarean; if anti-Xa levels are assayed, a test should be chosen that does not use exogenous antithrombin, targeting a peak 4 hours after administration of 0.5 to 1.0 UI/mL: high dose LMWH (50, 75 or 100% of full weight-adjusted dose).^45^

ACCP: low risk of recurrence: clinical observation;

Moderate to high risk without use of long-term anticoagulation: prophylactic or intermediate doses of LMWH.^46^

Postpartum

ACOG: prophylactic or intermediate doses of LMWH, UFH, or anticoagulation with VKA for 4 to 6 weeks.^44^

SOGC: prophylactic doses of UFH or LMWH (preferable) for 6 weeks.^43^

RCOG: LMWH, 50, 75 or 100% of full weight-adjusted dose for 6 weeks or until resumption of oral anticoagulation.^45^

ACCP: prophylactic or intermediate doses of LMWH or anticoagulation with VKA.^46^

###### Antiphospholipid antibody syndrome (APS)

Antepartum

ACOG: anticoagulation with heparin for entire pregnancy.^44^

SOGC: intermediate or therapeutic doses of UFH or LMWH (preferable).^43^

RCOG: management should involve a physician who is a specialist in anticoagulation or thrombosis during pregnancy: prophylaxis with high doses of LMWH (50, 75 or 100% of full weight-adjusted dose).^45^

ACCP: low risk of recurrence: clinical observation;

Moderate to high risk of recurrence without use of long-term anticoagulation: prophylactic or intermediate doses of LMWH.^46^

Postpartum

ACOG: 6 weeks of anticoagulation with heparin.^44^

SOGC: prophylactic doses of UFH or LMWH (preferable) for 6 weeks.^43^

RCOG: high doses of LMWH (50%, 75% or 100% of full weight-adjusted dose) until resumption of oral anticoagulation.^45^

ACCP: prophylactic or intermediate doses of LMWH or anticoagulation with VKA.^46^

##### Previous VTE associated with a transitory risk factor unrelated to estrogen, with no known thrombophilia

Antepartum

ACOG: clinical observation.^44^

SOGC: prophylactic doses of UFH or LMWH (preferable).^43^

RCOG: if the VTE was provoked by major surgery, with no other risk factors, thromboprophylaxis with LMWH can be started at 28 weeks, if there are no other risk factors; if the original VTE was related to transitory risk factors other than major surgery, LMWH should be administered for the entire pregnancy.^45^

ACCP: low risk of recurrence: clinical observation.^46^

Postpartum

ACOG: anticoagulant therapy postpartum.^44^

SOGC: prophylactic doses of UFH or LMWH (preferable) for 6 weeks.^43^

RCOG: prophylactic doses of LMWH or anticoagulation with VKA for at least 6 weeks.^45^

ACCP: prophylactic or intermediate doses of LMWH or anticoagulation with VKA for 6 weeks, if there is no C or S protein deficiency.^46^

##### Previous VTE associated with pregnancy or estrogen

Antepartum

ACOG: prophylactic doses of LMWH or UFH.^44^

SOGC: prophylactic doses of UFH or LMWH (preferable).^43^

RCOG: thromboprophylaxis with LMWH.^45^

ACCP: moderate to high risk of recurrence without use of long-term anticoagulation: prophylactic or intermediate doses of LMWH.^46^

Postpartum

ACOG: anticoagulant therapy postpartum.^44^

SOGC: prophylactic doses of UFH or LMWH (preferable) for 6 weeks.^43^

RCOG: prophylaxis with LMWH or anticoagulation with VKA for at least 6 weeks.^45^

ACCP: prophylactic or intermediate doses of LMWH or anticoagulation with VKA, for 6 weeks, if there is no C or S protein deficiency.^46^

##### Previous unprovoked VTE

Antepartum

ACOG: prophylactic doses of LMWH or UFH.^44^

SOGC: prophylactic doses of UFH or LMWH (preferable).^43^

RCOG: prophylactic doses of LMWH.^45^

ACCP: moderate to high risk of recurrence of VTE without use of long-term anticoagulation: prophylactic or intermediate doses of LMWH.^46^

Postpartum

ACOG: anticoagulant therapy postpartum.^44^

SOGC: prophylactic doses of UFH or LMWH (preferable) for 6 weeks.^43^

RCOG: prophylactic doses of LMWH or AVK for at least 6 weeks.^45^

ACCP: prophylactic or intermediate doses of LMWH or anticoagulation with VKA for 6 weeks, if there is no C or S protein deficiency.^46^

##### Two or more episodes of VTE without long-term anticoagulation

Antepartum

ACOG: prophylactic or therapeutic doses of LMWH or UFH.^44^

SOGC: prophylactic doses of UFH or LMWH (preferable).^43^

RCOG: management by a physician who is a specialist in thrombosis during pregnancy: high doses of LMWH (50%, 75% or 100% of weight-adjusted dose).^45^

ACCP: moderate to high risk of recurrence without use of long-term anticoagulation: prophylactic or intermediate doses of LMWH.^46^

Postpartum

ACOG: anticoagulation postpartum for 4 to 6 weeks.^44^

SOGC: prophylactic doses of UFH or LMWH (preferable) for 6 weeks.^43^

RCOG: high doses of LMWH (50%, 75% or 100% of full weight-adjusted dose) for 6 weeks.^45^

ACCP: prophylactic or intermediate doses of LMWH or anticoagulation with VKA for 6 weeks, if there is no C or S protein deficiency.^46^

##### Two or more episodes of VTE with use of long term anticoagulation

Antepartum

ACOG: therapeutic doses of LMWH or UFH.^44^

SOGC: prophylactic doses of UFH or LMWH (preferable).^43^

RCOG: women should be warned of the risks to the fetus of VKA and counseled to stop this medication and change to LMWH as soon as pregnancy is confirmed (the ideal is 2 weeks after a late period and before 6 weeks of pregnancy): high doses of LMWH (50%, 75% or 100% of full weight-adjusted dose).^44^

ACCP: when long-term VKA is being used and if the patient is a candidate for substitution with LMWH, it is suggested that pregnancy tests are conducted frequently and VKA is only substituted with LMWH when a pregnancy is confirmed. Adjusted doses or 75% of the therapeutic dose of LMWH are recommended.^46^

Postpartum

ACOG: resume long-term anticoagulation.^44^

SOGC: resume long-term anticoagulation.^43^

RCOG: high doses of LMWH (50%, 75% or 100% of full weight-adjusted dose) for 6 weeks until resumption of oral anticoagulation. Use of VKA can be resumed for women who were on long-term anticoagulation with this drug when the risk of bleeding has reduced, usually 5 to 7 days postpartum.^45^

ACCP: it is suggested that resumption of long-term anticoagulation is preferable to administration of prophylactic doses of LMWH.^46^

#### Prevention of VTE associated with caesarean delivery

A Cochrane systematic review concluded that there is not enough evidence for post-caesarean thromboprophylaxis because of the small number of studies and different comparison criteria.^47^ While the risk of VTE associated with caesarean is low,^15^^,^ 48 ^-^ 50 when it is present in combination with other risk factors, the rate of VTE occurrence becomes significant and thromboprophylaxis should be initiated.^43^^-^ 46

ACOG: intermittent pneumatic compression (IPC) before caesarean if the patient is not on thromboprophylaxis.^44^

SOGC: postpartum pharmacological prophylaxis is recommended in the following situations: previous VTE; high-risk thrombophilia (APS, antithrombin deficiency, homozygosity for factor V Leiden or G20210A prothrombin gene mutation, or combined thrombophilias), strict bed rest for 7 days or more before delivery, blood loss of at least 1 L during peripartum or postpartum, transfusion of blood products, postpartum surgery, and infection during peripartum or postpartum.^43^ Use of pharmacological prophylaxis should be considered in the event of two or more of the following: BMI ≥ 30 kg/m^2^ at first prenatal consultation, smoking > 10 cigarettes/day, pre-eclampsia, fetal growth restriction, placenta previa, emergency caesarean, blood loss of at least 1 L during peripartum or postpartum or transfusion of blood products, low risk thrombophilia (C or S protein deficiency, heterozygosity for factor V Leiden or G20210A prothrombin gene mutation), maternal cardiac disease, systemic lupus erythematosus, sickle-cell anemia, intestinal inflammatory disease, varicose veins in lower limbs, gestational diabetes, premature delivery, still birth, or three or more of the following risk factors: age > 35 years, parity ≥ 2, any type of assisted reproductive technology, multiple pregnancy, placental abruption, premature rupture of membranes, elective caesarean, or maternal cancer. Women with persistent risk factors should be given thromboprophylaxis for a minimum of 6 weeks postpartum; women with transitory risk factors during antepartum or peripartum should be given thromboprophylaxis until hospital discharge or up to 2 weeks after birth.^43^

RCOG: emergency caesarean, 10 days after delivery with prophylactic doses of LMWH; for all other caesarean patients, consider 10 days of LMWH at prophylactic doses if there are other risk factors.^45^

ACCP: in the absence of additional risk factors, no prophylaxis should be prescribed beyond early mobilization; in cases with one major risk factor or ≥ two minor risk factors (or one minor factor if caesarean was an emergency), prophylaxis with LMWH after delivery is suggested while the patient is still in hospital (if there are contraindications to anticoagulation, use mechanical prophylaxis with elastic stockings or IPC); in extremely high-risk cases with additional risk factors that persist into puerperium, combine LMWH with elastic stockings and/or IPC; selected high-risk patients with additional risk factors that persist into puerperium should be given up to 6 weeks extension of prophylaxis after hospital discharge.^46^

## CONCLUSIONS

The recommendations suggested here could be subject to individual variations between different patients and are intended to inform and not to substitute the clinical judgment of the physician, who is ultimately responsible for determination of the appropriate treatment for each individual patient. Notwithstanding, with appropriate prophylactic management, the incidence of VTE in pregnant women can be reduced, thereby preventing its acute and chronic complications.
